#  Diagnostic Accuracy of Frozen Section of Central Nervous System Lesions: A 10-Year Study 

**Published:** 2015

**Authors:** Maliheh KHODDAMI, Ali AKBARZADEH, Afshin MORDAI, Farahnaz BIDARI - ZEREHPOUSH, Hamid ALIPOUR, Sara SAMADZADEH, Bijan ALIPOUR

**Affiliations:** 1 Pediatric Pathology Research Center, Shahid Beheshti University of Medical Sciences (SBUMS), Tehran, Iran.; 2 Department of Pathology, Shahid Beheshti University of Medical Sciences (SBUMS), Tehran, Iran.; 3 Department of Surgery, Imam Reza Hospital, Kermanshah University of Medical Sciences (KUMS), Kermanshah, Iran.; 4 Department of Pediatrics, Imam Reza Hospital, Kermanshah University of Medical Sciences (KUMS), Kermanshah, Iran.; 5 Research Center for Immunodeficiencies, Children’s Medical Center, Tehran University of Medical Sciences (TUMS), Tehran, Iran.

**Keywords:** CNS lesions, Frozen section, Intraoperative consultation, Accuracy

## Abstract

**Objective:**

Definitive diagnosis of the central nervous system (CNS) lesions is unknown prior to histopathological examination. To determine the method and the endpoint for surgery, intraoperative evaluation of the lesion helps the surgeon. In this study, the diagnostic accuracy and pitfalls of using frozen section (FS) of CNS lesions is determined.

**Materials & Methods:**

In this retrospective study, we analyzed the results of FS and permanent diagnoses of all CNS lesions by reviewing reports from 3 general hospitals between March 2001 and March 2011.

**Results:**

273 cases were reviewed and patients with an age range from 3 to 77 years of age were considered. 166 (60.4%) had complete concordance between FS and permanent section diagnosis, 83 (30.2%) had partial concordance, and 24 cases (9.5%) were discordant. Considering the concordant and partially concordant cases, the accuracy rate was 99.5%, sensitivity was 91.4%, specificity was 99.7%, and positive and negative predictive values were 88.4% and 99.8%, respectively.

**Conclusion:**

Our results show high sensitivity and specificity of FS diagnosis in the evaluation of CNS lesions. A Kappa agreement score of 0.88 shows high concordance for FS results with permanent section. Pathologist’s misinterpretation, small biopsy samples (not representative of the entire tumor), suboptimal slides, and inadequate information about tumor location and radiologic findings appear to be the major causes for these discrepancies indicated from our study.

## Introduction

Frozen section (FS) diagnosis provides useful information for surgery as it guides a surgeon with appropriate therapeutic decision-making on most occasions. In patients with central nervous system (CNS) lesions, rapid intraoperative diagnosis helps the surgeon to determine the best procedure and the endpoint of the operation (1,2). Many studies have confirmed the accuracy of FS diagnosis for assessment of the CNS lesions, with acceptable sensitivity (3-8). However, no published information is available in Iran concerning its diagnostic accuracy and pitfalls. We retrospectively study the reliability of FS diagnosis for CNS lesions in three hospitals over a 10- year period. 

## Materials & Methods

In this retrospective study, we reviewed the reports of all CNS lesions from March 2001 to March 2011 in the departments of Pathology of three general hospitals that are referral centers for neurosurgical cases. There were 273 CNS cases. During intraoperative consultation, fresh specimens were examined grossly for size and consistency. Based on sample size, representative sections were taken by an attending pathologist. Touch or squash preparations were also used based on tissue consistency. The tissue was frozen in a cryostat (-25°C) and 6–7μ sections were prepared and stained with Hematoxylin and Eosin (H&E). The slides were examined under a light microscope by one or more general pathologists. After formalin fixation, additional representative sections were taken for paraffin embedding and routine H&E staining. The definitive diagnosis was made after a thorough microscopic examination of all slides. In these institutions, few pathologists have special training in the field of neuropathology and the lesions were usually evaluated by general pathologists with 5–25 year experience in the field. Histological diagnosis was made according to the criteria set forth in pathology textbooks for diagnosis of CNS lesions (9,10). For histological typing of tumors, WHO recommendations (10) were followed. 

The reports of frozen and permanent sections were evaluated according to the status of benignancy, malignancy, and histological type of the lesions. The results from the permanent sections were used as a gold standard. A definitive diagnosis was deferred to paraffin section analysis when the sample was too small for proper evaluation. 

The overall sensitivity, specificity, false positive (FP), false negative (FN), positive predictive value (PPV), and negative predictive value (NPV) of FS were determined according to the type of the lesions and the accuracy of histological tumor types. 

The cases were graded into three degrees of diagnosis concordance to clarify the accuracy of intra operative diagnosis as follows: 

1- FS diagnosis was exactly the same as the final diagnosis (complete concordance); 

2 - FS diagnosis was not incorrect but was too broad to qualify as complete concordance (partial concordance); or, 

3- FS diagnosis was incorrect and different from the final diagnosis (no concordance or discordant). 

All statistical analyses were performed using SPSS (ver 20). To describe data, we used mean, standard deviation, median, and percent. To evaluate the agreement of the methods, we utilized sensitivity, specificity, NPV, and PPV. 

In this study, no ethical issues were involved as only pathology reports were reviewed retrospectively and all patients were anonymous. The articles used as references are valid and the information taken are reported unchanged. 

## Results

During the study period, 273 cases were retrieved from three hospitals. The ages of the patients ranged from 3–77 years (mean age 35.1 years old); 167 (61.1%) were male and 106 (38.9%) were female. 

Out of 273 cases, 166 cases (60.4%) were completely concordant, 83 cases (30.2 %) had partial concordance, and 24 cases (9.5%) were discordant. Considering concordant and partially concordant cases, the diagnostic accuracy was 99.5 %. The sensitivity and specificity were 91.4% and 99.7%, respectively. The positive predictive value was 88.4% and negative predictive value was 99.8%. A Kappa agreement score of 0.88 shows high concordance of FS results with permanent sections. 

The majority of tumors (53%) were located in parietal lobe (17%), sellar region (14%), frontal lobe (12%), and temporal lobe (10%). Tables 1 and 2 summarize the number of cases according to the type of lesion and degree of concordance. Table 3 summarizes discordant cases. The partially concordant cases (83 cases) included those in which FS diagnoses were not incorrect but was too broad to qualify as complete concordance. Examples of these cases are as follows: FS: glial tumor, permanent: astrocytoma or oligodendroglial tumor or ependymoma; FS: low grade glial tumor, permanent: ganglioglioma; FS: non-germ cell tumor, permanent: pineocytoma; FS: Positive for malignancy, permanent: clear cell tumor or metastatic tumor; FS: benign spindle cell tumor, permanent: meningioma; FS: malignant round cell tumor, permanent: lymphoma; FS: epidermoid cyst, permanent: dermoid cyst; FS: inflammatory process, permanent: granulomatous inflammation. Deferred cases (due to small size of specimen) included one pilocytic astrocytoma, one pineocytoma, one hemangioma, and one ependymoma. 

## Discussion

Intraoperative consultation (FS) can provide surgeons with a primary diagnosis that is helpful to decide a subsequent surgical approach. It can inform the surgeon as to whether the biopsy is taken from the appropriate area and the adequacy of the specimen that is important to the pathologist to make a final diagnosis on the permanent sections can be determined (2). The reported diagnostic accuracy of CNS FS diagnosis is greater between 85–90% in previous studies with a 92–97% agreement degree between final diagnosis with FS and the permanent section. The majority of discordancies between FS and the permanent diagnoses were seen in ependymoma, glioblastoma, metastatic tumors, oligodendroglioma, meningioma, and astrocytoma (3,4). 

Thomas et al had less than 3% discordancy among 2,156 cases during an 8-year period. Approximately 80% of the discrepant cases were spindle cell lesions, astrocytoma versus oligodendroglioma, lymphoma, reactive versus neoplastic process, and tumor overgrading (5). A French study on1, 315 cases found 96.6% concordance between FS and permanent diagnoses. Most discrepancies were in gliomas, hemangioblastomas, and metastatic tumors (3). Diagnostic accuracy was 92.4% in Talan Hernilovic (6) and 95% in Roessler (7) with 89% complete concordance among 4,172 patients. The most accurate FS diagnoses were made in cases of meningioma (97.9%), metastasis (96.3%), and glioblastoma(95.7%) (7). In a referral center in Pakistan, the diagnostic accuracy was 88.9% in CNS neoplasms for 171 cases (8). Our results showed a high accuracy percentage for frozen sections in the diagnosis of CNS lesions (99.5%), when concordant and partially concordant cases were included and considering partially concordant cases were correct but not as accurate as completely concordant diagnoses. In our study, 166 of 273 cases were in complete concordance. Most of our discordant results were in astrocytic tumors (5 cases), followed by pituitary adenoma, germ cell tumors, ependymal tumors, schwannoma, neurocytoma, embryonal tumors, and metastatic tumors. A total of 84% (21 cases) of meningiomas were diagnosed correctly with FS analysis. 

**Table 1:** General Categories of Studied Cases

**Type of diagnosis**	**No. of cases**	**Concordance**		
		**No (%)**	**Partial**	**Complete**
**Total**	**273**	**26 (9.5)**	**83 (30.2)**	**166 (60.4)**
**Astrocytic tumors**	97	6(6.2)	48 (49.5)	43 (44.3)
**Tumors of the meninges**	25	0 (.0)	4 (16.0)	21 (84.0)
**Ependymal tumors**	20	2 (10.0)	3 (15.0)	15 (75.0)
**Tumors of cranial and paraspinal nerves **	13	2 (15.4)	1 (7.7)	10 (76.9)
**Embryonal tumors**	12	2 (16.7)	0 (.0)	10 (83.3)
**Oligodendroglial tumors**	10	0 (.0)	7 (70.0)	3 (30.0)
**Metastatic tumors**	10	2 (20.0)	1 (10.0)	7 (70.0)
**Mesenchymal tumors**	8	3 (37.5)	1 (12.5)	4 (50)
**Lymphoid and hematopoietic neoplasms**	7	1 (14.3)	2 (28.6)	4 (57.1)
**Oligoastrocytic tumors**	6	0 (.0)	3 (50.0)	3 (50.0)
**Tumors of the pineal region**	6	0 (0)	3 (50.0)	3 (50.0)
**Neuronal and mixed neuronal-glial tumors**	5	1 (20.0)	3 (60.0)	1 (20.0)
**Germ cell tumors**	5	3 (60.0)	0 (.0)	2 (40.0)
**Choroid plexus tumors**	4	1 (25.0)	0 (.0)	3 (75.0)
**Tumors of the sellar region**	4	1 (25.0)	0 (.0)	3 (75.0)
**Chordoma**	3	0 (.0)	0 (.0)	3 (100.0)
**Undifferentiated tumor**	3	0(0)	1 (33.3)	2 (66.7)
**Other neuroepithelial tumors**	2	0 (.0)	1 (50.0)	1 (50.0)
**Optic nerve glioma**	2	0 (.0)	0 (.0)	2 (100.0)
**Eosinophilic granuloma**	1	0 (.0)	0 (.0)	1 (100.0)
**Clear cell neoplasm**	1	0 (.0)	1 (100.0)	0 (.0)
**Malignant neoplastic lesion composed of blastic and epithelial component**	1	0 (.0)	0 (.0)	1 (100.0)
**Malignant neoplasm**	1	0 (.0)	0 (.0)	1 (100.0)
**Malignant epithelioid tumor**	1	0 (.0)	1 (100.0)	0 (.0)

**Table 2:** Continuation of Table 1

**Type of diagnosis**	**No. of cases**	**Concordance**		
		**No (%)**	**Partial**	**Complete**
**Pituitary adenoma**	14	2 (14.3)	1 (7.1)	11 (78.6)
**Necrotic tissue**	3	0 (.0)	0 (.0)	3 (100.0)
**Compatible with tuberculosis**	2	0 (.0)	1 (50.0)	1 (50.0)
**Inadequate specimen**	2	0 (.0)	1 (50.0)	1 (50.0)
**Granulomatous reaction**	2	0 (.0)	1 (50.0)	1 (50.0)
**Inflammatory process**	1	0 (.0)	0 (.0)	1 (100.0)
**Extramedullary hematopoiesis**	1	0 (.0)	0 (.0)	1 (100.0)
**Giant cell lesion most probably ** **chondromyxoid fibroma**	1	0 (.0)	0 (.0)	1 (100.0)
**Dermoid cyst**	1	0 (.0)	1 (100.0)	0 (.0)
**Hydatid cyst**	1	0 (.0)	0 (.0)	1 (100.0)

In addition to histologic similarities between different lesions, limited sampling, suboptimal slide preparation, pathologist expertise, and lack of communication between pathologists and surgeons are all important factors for inaccurate FS diagnoses. Sometimes it is difficult to diagnose cytoplasmic processes and fibrillary patterns in FS slides, which makes it impossible to differentiate glial tumors from carcinoma, lymphoma, and melanoma. Incorrect FS diagnosis of astrocytomas in some cases could be due to the thickness of the cuts and technical problems with staining which results in the disruption of cellular morphology (9). The diagnosis of ependymoma by FS is easy; however, sometimes because of fewer cells and more fibrillary tissues in the samples, it is misdiagnosed as astrocytoma (2 of our cases) (9). Pituitary adenoma could also be misdiagnosed as malignant round tumors in FS due to round monomorphic cells in the pituitary adenoma, as was the case in one of our patients. Medulloblastoma sometimes shows the presence of perivascular rosettes, which makes it difficult to differentiate it from ependymoma (also, a case from our study) (9). 

Conclusion: The role of FS in the intraoperative consultation is important. Our results show a high percentage of accuracy in the intraoperative diagnosis of CNS lesions. Appropriate communication between pathologists and neurosurgeons with adequate information about the radiologic findings is helpful to minimize FS misdiagnosis. Although pathologist misinterpretation, small biopsy samples, suboptimal slides, and inadequate information about tumor location and radiologic findings were major causes of discrepancies in our study, an experienced pathologist could still be relied upon for intraoperative consultation of CNS lesions. 

**Table 3:** Details of Discordant Diagnoses

Permanent Diagnosis	Frozen diagnosis	No. of cases
ASTROCYTIC TUMOURS		
Glioblastoma multiforme Diffuse astrocytoma Diffuse astrocytoma Astrocytic tumor Astrocytic tumor	Ependymal tumour Inflammatory process Gliosis Tumoral lesion Hematopoietic neoplasm	1 1 1 1 1
NEURONAL AND MIXED NEURONAL–GLIAL TUMORS		
Neurocytoma Neurocytoma	Ependymoma Malignant round cell tumor	1 1
EMBERYONAL TUMORS		
Desmoplastic medulloblastoma	Ependymoma	1
TUMORS OF CRANIAL AND PARASPINAL NERVES		
Schwannoma Schwannoma	Glial neoplasm Hypercellular hyalinized tissue	1 1
MESENCHYMAL TUMOURS, NOT OTHERWISE SPECIFIED	Malignant round cell tumor	1
HEMATOPOIEIC NEOPLASM		
Plasma cell tumor	Pituitary adenoma	1
GERM CELL TUMOURS Germinoma Mixed germ cell tumor	Diffuse astrocytoma Craniopharyngioma	1 1
METASTATIC TUOMOURS		
Metastatic tumor Metastatic tumor	Inflammatory process Hemorrhagic vascular lesion	1 1
PITUITARY ADENOMA		
Pituitary adenoma Pituitary adenoma	Craniopharyngioma Malignant round cell tumor	2 1
OTHERS		
Ependymal tumor Choroid plexus tumor Chordoma Subependymoma	Astrocytic tumor Ependymal tumor Immature mesenchymal tissue, possibly teratoma Neurocytoma	2 1 1 1

## Notes

**Funding Support: **None
